# The Discrepancy between Clove and Non-Clove Cigarette Smoke-Promoted *Candida albicans* Biofilm Formation with Precoating RNA-aptamer

**DOI:** 10.12688/f1000research.52266.2

**Published:** 2021-07-12

**Authors:** Boy Muchlis Bachtiar, Basri A. Gani, Astri Deviana, Nastiti Rilo Utami, Anissa Dien Andriyani, Endang Winiati Bachtiar

**Affiliations:** 1 Department of Oral Biology, Faculty of Dentistry Universitas Indonesia, Jakarta, DKI, 10430, Indonesia; 2 Oral Biology Department, Faculty of Dentistry Universitas Syah Kuala, Banda Aceh, Nagroe Aceh, 23111, Indonesia; 3 Oral Biology Laboratory, Faculty of Dentistry Universitas Indonesia, Jakarta, Indonesia, 10430, Indonesia

**Keywords:** Cigarette Smoke, Candida albicans, Biofilm, RNA-aptamer, ALS3 and HWP1.

## Abstract

This study explores the influence of precoating aptamer (Ca-apt1) on *C. albicans* viability while the fungus was growing in the presence of exposing condensed cigarette smoke (CSC), prepared from clove (CCSC) and non-clove (NCSC) cigarettes, for 48 h. Using qPCR, we found that mRNA expression of adhesion-associated genes ( *ALS3 and HWP1*) was impaired by precoating *C. albicans* yeast cells with the aptamer. Conversely, the gene transcription was upregulated when aptamer-uncoated yeast was pre-treated with either CSC. In addition, by analysing the result of MTT ([3-(4,5-dimethyl-2-thiazolyl)-2,5-diphenyl-2H-tetrazolium bromide] assay, we found that the presence of added CCSC or NCSC in growth medium for 48 h was significantly enhanced *C. albicans* biofilm development. However, the presence of precoated aptamer was significantly impaired biofilm development accelerated by the NCSC. The inhibitory effect of the Ca-apt1 was not dependent on the precoated aptamer (1 and 10%). Interestingly, we noted that the enhancer effect of treated CCSC was no longer effective when the yeast had been precoated with 10% aptamer tested. Additionally, light microscopy analysis revealed that precoating aptamer alleviates morphological changes of *C. albicans* (from yeast to hypha formation) that are enhanced by adding CCSC or NCSC in the growth medium.

In conclusion, these results suggest that administration on Ca-ap1 exhibits a significant protective effect on CSC-induced biofilm formation by *C. albicans*.

## Introduction

*Candida albicans* is a normally harmless inhabitant of the oral cavity. However, unlike other fungal pathogens that exist primarily in either yeast or hyphal forms, *C. albicans* is an opportunistic pathogen. The fungus’s behaviour correlates with its ability to grow in distinct morphogenic states, including budding yeast or blastospores, pseudo hyphae, and true hyphae ^1– 3^. This morphogenic transition from yeast to hypha form is important for the pathogenesis of *C. albicans*, is dependent on how the fungus cell responds to the environmental cues ^4^. As shown in the literature, cigarette smoke is one factor that can aid and accelerate this transformation ^5– 7^, and there is a clear association between oral candidiasis and smoking habit ^8^. In this regard, the presence of nicotine in cigarette smoke promotes *C. albicans* growth rate and adhesion, in which, a higher level of expression of genes related to adherence, such as *ALS3* and *HWP1* have been reported ^9^. 

Smoking is a social habit in many countries ^10^, and it is an important public health problem, including in Indonesia ^11^. However, despite the extensive research exploring the deleterious role of a smoking habit on oral microorganisms, there is little information about the effect of non-conventional tobacco, i.e., clove cigarettes, on the virulence attributes of *C. albicans*. By searching the literature, we found that, like conventional cigarettes, the clove cigarette, commonly known as kretek, is the most popular ^12^ in Indonesia. It is also a source of numerous toxicants, and they have a potential implication in the oral ecosystem ^13^, which may also influence oral *Candida* pathogenicity ^14^. The unique aspect of kretek is the dried clove buds it contains ^15^ which have never been identified in a conventional cigarette. The involvement of clove cigarette smoke condensate (CCSC) on the biofilm formation of *C. albicans* remains obscure. Given that cigarette smoke contains many toxicants ^13^, it is important to explore the involvement of CCSC on the growth and morphogenesis of *C. albicans*, as this fungus is the most implicated oral pathogen in the clinical setting.

During the last several years, aptamers, either single strain DNA or RNA, and against different microorganism species, have become the focus of growing interest. In an earlier study, we reported an anti- *Candida* activity of an RNA-aptamer (Ca-apt1) against the fungus while growing as biofilm ^16^. Here, we evaluated the aptamer's beneficial properties against *C. albicans* biofilm formation induced by cigarette smoke. Particularly, the aptamer's ability to restrict the transition of fungus phenotype from yeast to hyphal form.

## Methods

### *C. albicans* and *RNA Aptamer used*

In this study, we used a clinical isolate that we collected previously from the denture surface of denture wearer subject ^17^ and selected by using CHROMAgar ^18^, while the reference strain ( *C. albicans* ATCC 10231), which was used as a targeted aptamer ligand ^16^, was needed for the evaluation and validation of this experimental study. Briefly, all microorganisms were taken from stock cultures frozen in 15% glycerol at -80°C and sub-cultured onto yeast peptone agar plates (1% yeast extract, 2% peptone, 2% glucose;YPD) or when indicated in yeast nitrogen base medium, supplemented with 50 mM glucose (YNB).

The synthetic oligonucleotide used in this study was RNA-aptamer (Ca-apt 1) that was obtained from the systemic evolution of ligands by exponential enrichment (SELEX) method ^16^. 

### Preparation of cigarette smoke condensates

**Figure 1.**
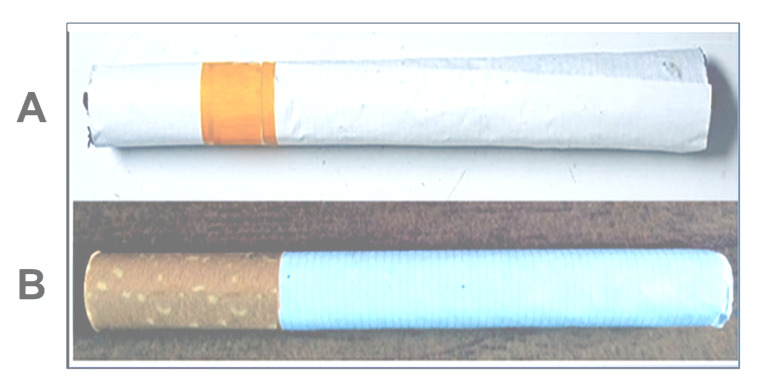
Cigarettes used to prepare condensate cigarette smoke. **A** and **B** are Clove and non-clove cigarette, respectively.

In this study, condensed smoke cigarettes (CSC) were generated from Indonesia's non-filtered clove cigarette (kretek) and imported non-clove cigarettes ( Figure 1) that we purchased from a local tobacco outlet. The CSC, either from a clove cigarette (CCSC) or a non-clove cigarette (NCSC), was prepared by smoking five cigarettes that were smoked to an in-house smoking device ^5^ and concentrated in 100 mL of 0.09% Sodium Chloride solution. The resulting condensate smoke solution was further sterilized by filtration through a Millipore filter (0.22 µm). The influence of CCSC/ NCSC-containing YNB (pH was adjusted to 7.2) on the viability of aptamer-precoated *C. albicans* in the preformed biofilm was assessed colorimetric (MTT) assay and kept at 4°C until use.

### Analysis of mRNA expression of *ALS3* and *HWP1* and microscopic image in *C. albicans* with and without precoating aptamer

To determine that the inhibition effect of the tested aptamer in the early biofilm development stage was independent from the biofilm biomass maturation, we separated biofilm development into adhesion and growth phases. Initially, by analysing the mRNA expression of adhesion-associated genes ( *ALS3* and *HWP1*), we wanted to confirm if the inhibition event was due to the lessened ability of *C. albicans* to switch its phenotype from yeast to hypha form. To do this, the test aptamer was first preincubated with *C. albicans* yeast cells before the fungus was inoculated into microplate wells. Further, we did an analysis of mRNA expression of adhesion-associated genes ( *ALS3* and *HWP1*) after 90 min incubation time. The transcription level of these genes was also measured after *C. albicans* was exposed with either cigarette smoke, but without precoating aptamer. This was done to evaluate CSC's involvement in the transition *C. albicans* morphogenetic, from yeast or blastospore to hyphae form. All the procedures used as described in a previous study ^17^. Briefly, RNA isolation, purification, and reverse transcription of cDNA were conducted using TRIzol™ Reagent (Invitrogen Life Technologies, Carlbad, California, United States) followed by reverse transcription using the TaqMan® Reverse Transcription Reagents (Applied Biosystems, Waltham, Massachusetts, USA). The resulting cDNA (1 µg) was amplified by qPCR with specific primers used in our previous study ^19^. The qPCR analysis was performed in ABI StepOnePlus™ Real-Time PCR Systems (Applied Biosystems) with Platinum™ SYBR™ Green qPCR SuperMix-UDG (Invitrogen). The qPCR cycling conditions consisted of a 10-minute initial denaturation at 95°C followed by 40 PCR cycles of 15 seconds at 95°C and one minute at 60°C. The formula of fold change 2 ^-ΔΔCt ^was used to calculate the relative mRNA expression, which was compared with that of the housekeeping gene, 18S rRNA. The mRNAs gene ( *ALS3* and *HWP1*) expressed by *C. albicans* without bound aptamer or without CSC exposure were used as a control, set at one.

### Biofilm formation of *C. albicans*

To test the effectivity of CSC, with and without precoating aptamer, on *C. albicans* biofilm formation, we used a biofilm assay that was performed as previously described ^19^. Briefly, 100 µL containing 1.8 X 10 ^*5*^ yeast cells of *C. albicans* (counted by using a haemocytometer), from overnight culture in YPD broth was aliquot into 96-well microtiter plates containing mixture of 70% of fresh yeast nitrogen base (YNB; sigma-Aldrich) and 30% condensate smoke (vol/vol). The pH of the mixture was adjusted to neutrality (7.0) using 1M NaOH. This was done to define that the pH of the mixture does not play a role in modulating biofilm formation.

The aptamer was prepared by separating it in two different concentrations (1ng/µL and 10 ng/µL) in buffer ^16^, prior to being added into separate wells, and the plates were incubated at 37°C in 5% CO2 in air for 90 min with gentle shaking. *Candida albicans* biofilm with mix medium added instead of aptamer was used as a negative control. To promote biofilm formation, the adhered cells were washed twice with sterile Phosphate Buffer Saline (PBS), *C. albicans* were further grown in a medium (150 µL) containing growth medium without aptamer. The culture period was further lengthened to 48-h. The extent of biofilm formation estimated using the semi-quantitative MTT (3-(4,5-dimethylthiazol-2,5-diphenyltetrazolum bromide) reagent (Sigma-Aldrich, St. Louis, MO, USA). The absorbance was measured spectrophotometrically at 450 nm with 620 nm as the reference wavelength for this assay. The results expressed as OD450/620, and were correlated with cellular metabolic activities within the biofilm. The assay was done in triplicate, repeated two times independently. Moreover, the percentage of fungal damage was calculated based on data obtained in MTT assay, using the formula: 100X [(1- OD _CandidaCSC+apt _/ OD _Candida alone_)]. Biofilm formation on the bottom of microtiter well plates was qualitatively observed using an inverted light microscope.

### Analysis of microscopy image

Following incubation for 90 min or 48-h, *C. albicans* cultures were observed microscopically and its morphology images were analysed qualitatively.

### Statistical analysis

Statistical analysis was performed with GraphPad Prism ^TM ^(version 9.00) software (GraphPad Software, Inc., San Diego, California, USA). The two-way analysis of variance (Two-way ANOVA) with Geisser-Greenhouse correction was used to verify the significant level of response between and within groups comparison. Each experiment was carried out in triplicate wells and repeated at least twice, independently. A P < 0.05 value was considered statistically significant.

## Consent statement

Written informed consent was obtained from participants for use of their data.

## Ethical approval

The samples were collected, after informed consent was obtained from all the participants ^17^, in accordance with the approved protocol of the Bioethics Committee of the Faculty of Dentistry, Universitas Indonesia (protocol number 020950818). The protocol conformed to the criteria of the Helsinki Declaration and the good clinical practical guidelines of the International Council on Harmonization. 

## Results

### Evaluation of the mRNA levels of *ALS3* and *HWP1* in pre-coating aptamer *C. albicans*

**Figure 2.**
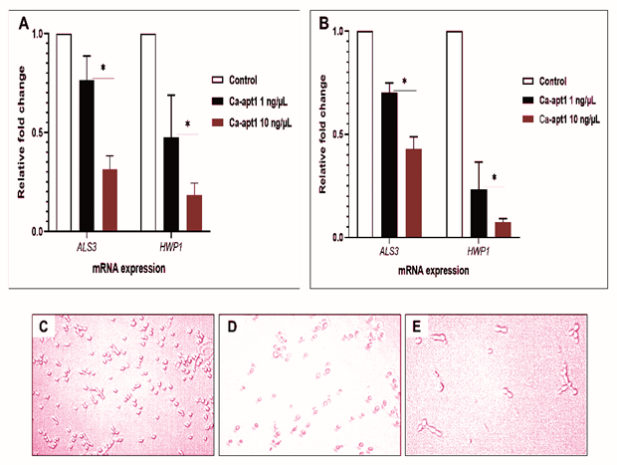
Induction of selected biofilm-associated genes by *C. albicans* following precoating with different concentrations of Ca-apt1. The upper panel shows the effect of precoating aptamer on *ALS3* and *HWP1* expressed by *C. albicans* in preformed biofilm (90 min incubation time) were analysed by qPCR. The mRNA expression levels were normalized relative to the control (yeast cells without aptamer precoating), which was set to one for each gene to determine the fold change in expression of genes in *C. albicans* clinical isolate ( **A**) and the ATCC 10231 ( **B**). The results are expressed as the mean and standard deviation (SD) of triplicate experiments and repeated two times independently. *Significantly higher downregulation in the expression of mRNA (P < 0.05) in the presence of precoating aptamer. The lower panel is representative of micrograph of *C. albicans* yeast cell (that only shown in clinical isolate) without precoating aptamer ( **C**), and the reduced germinated cells after incubating for 90 min with precoating aptamer 1 ng/μL ( **D**) and 10 ng/μL ( **E**). All images were captured by using light microscopic at X 200 magnification.

To understand the underlying relationship between precoating aptamer (Ca-apt1) and *C. albicans* morphogenesis at molecular level, we firstly compared the expression profile of two adhesion-associated genes ( *ALS3* and *HWP1*). To do this, the test aptamer was preincubated with *C. albicans* yeast cells before the biofilm was allowed to form. Further, we combined quantitative analysis of mRNA expression of these genes and qualitative microscopic images to describe the adhesion event. The result showed, that after 90 min, the expression of *ALS3* by the adherence cells were proportionally reduced by ≈ 8% and ≈ 4%, for aptamer concentration 1 ng/µL and 10 ng/µL, respectively. A similar trend in the expression of adhesion-associated genes was noted in either *C. albicans* strain used. We noted that the reduction of *HWP1* expression was higher than *ALS3* under different concentrations of Ca-apt1. In either *C. albicans* strain, this gene downregulated was > 50% ( Figure 2A and B). Further, the microscopic images show that at the attachment phase (90 min), the biofilm consisted of both yeast cells and blastospore that adhered to the surface, and those cells adhered to other cells attached to the surface ( Figure 2C–E).

### CCSC and NCSC promoted the transcription level of *ALS3* and *HWP1* by *C. albicans*

**Figure 3.**
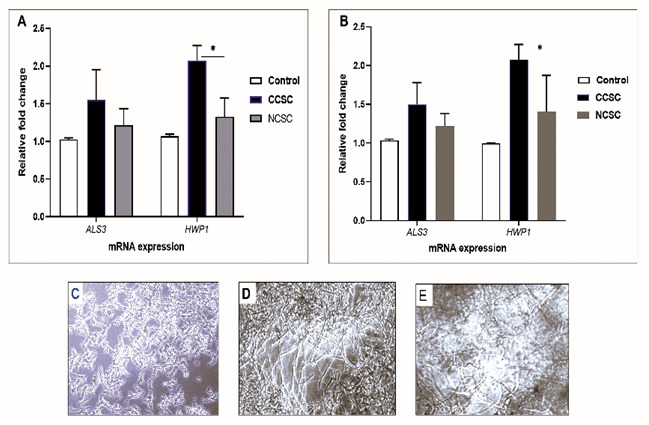
Effect of cigarette smoke condensate on the transcription level of selected *C. albicans* biofilm-associated genes. The qPCR analysis of *ALS3* and *HWP1* genes is shown after the preformed biofilm (90 min incubation) was treated by growth medium supplemented with different CSC (CCSC or NCSC). The mRNA level of each gene was normalized to that of 18S rRNA, while the expression level of the control (untreated biofilm) was set to one for each gene to determine the fold change in the expression of each targeted gene in clinical isolate ( **A**) and the ATCC strain ( **B**) of *C. albicans*. *Significantly higher upregulation of mRNA expression of *HWP1* induced by CCSC than NCSC (P < 0.05). Data are expressed as the mean and standard deviation (SD) of triplicates from two separate experiments. The CCSC and NCSC are clove and non-clove cigarettes smoke condensate, respectively. The lower panel shows the 48-h biofilm formation that only shown in clinical isolate; the control ( **C**) and those biofilms treated with CCSC ( **D**) and NCSC ( **E**), respectively. All the biofilms are visualized by using light microscopic images at X 200 magnification.

Next, we tested the mRNA expression of *ALS3* and *HWP1* on the biofilm formation promoted by either cigarette smoke condensate (CSC) after 48-h incubation time without precoating aptamer. As shown in Figure 3A and B, by comparing CCSC and NCSC, we observed that *Candida* adhesion was due to the presence of CCSC that subsequently increased the transcription level of hypha-associated gene ( *HWP1*), compared to the biofilm induced by NCSC-added growth medium (p< 0.05). For the adhesion-related gene ( *ALS3*), the transcription level was comparable when induced by either CCSC or NCSC-treated growth medium or growth medium only (control/ unexposed *C. albicans*). We noted that both *C. albicans* strain (clinical isolate and ATCC) showed a similar pattern. Figure 3C–E show the result provided by the microscopic analysis. After a 48-h time period, the hyphal form was more abundant in CCSC-treated growth medium than those in NCSC-treated biofilm.

### Precoated RNA aptamer inhibits *C. albicans* biofilm formation

**Figure 4.**
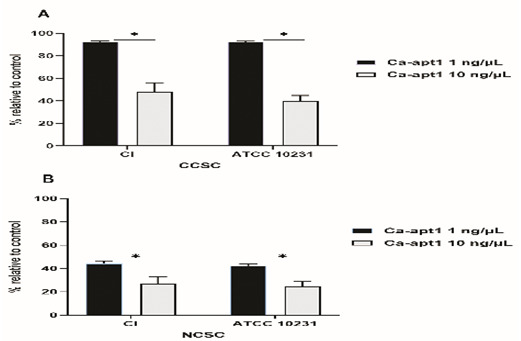
Effect of condensate smoke on preformed biofilm cells. The effect of CCSC ( **A**) and NCSC ( **B**) exposure on biofilm formation of *C. albicans*, which had been precoated with different concentration of Ca-apt1, evaluated after incubation for 48-h. The relative biofilm formation compared to biofilm without the precoating aptamer (control) was calculated. Data represent the mean and standard deviation (SD) of three biofilms grown on two separate occasions. Asterisks denote statistically significant differences between the effect of cigarette smoke condensate determined by MTT assay, p < 0.05. The CCSC and NCSC are clove and non-clove cigarette smoke condensate, respectively.

**Figure 5.**
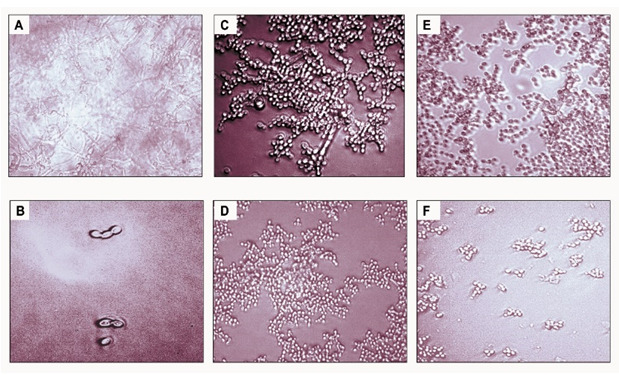
Light microscopy photomicrograph illustrating the different morphology of *C. albicans* with and without precoating aptamer (Ca-apt1) and grown for 48-h in the growth medium supplemented with cigarette smoke condensate (CSC). The pictures show biofilm of *C. albicans* derived from clinical isolate, without either precoating aptamer or added CSC ( **A**), *C. albicans* with precoated aptamer but untreated with either CSC/ CCSC or NCSC ( **B**), *C. albicans* precoated with 1% aptamer, CCSC ( **C**) and NCSC ( **E**), and 10% aptamer with CCSC ( **D**) and NCSC ( **F**). All microscopic image show at X 200 magnification.

Next, we tested the impact of different concentrations of precoating aptamer (1% and 10%) on *C. albicans* biofilm formation. For this, biofilms were developed in CCSC/NCSC-containing YNB (pH was adjusted to 7.2), and the quantification of biofilm cell (MTT assay) was evaluated after the biofilms reach the maturation time (48-h). This method was done, because we wanted to determine whether the precoating aptamer could adversely affect *C. albicans* biofilm formation after treating with the biofilm enhancers (CCSC or NCSC). Our data found that in comparison to the control, 1µg/ µL aptamer concentration of precoated yeast cells was sufficient to reduce biofilm cell, by about 10%, and 40 %, as observed in CCSC and NCSC-treated biofilm, respectively. At this maturation state, the viable cells detected within this biofilm were significantly reduced (p< 0.05), because of the increased aptamer concentration by 10% in the pre-adherence phase of the biofilm ( Figure 4A–B). Further, the effect of CCSC or NCSC on the transition of yeast to hypha morphology in pre-adhered cells was visualized microscopically. We observed that when the fungus cells were precoated with aptamer without exposed with CCSC or NCSC and incubated for 48-h, the pre-formed biofilms were dominated by blastospores, as compared to the control cells (uncoated cells), which showed that the adhered cells were dominated by dense hyphae form ( Figure 5 A–B). This qualitative effect was also dependent on the concentration of the aptamer tested ( Figure 5C–F).

## Discussion

The current study attempted to get further insight into clove cigarettes' effect on non-mammalian eukaryotes cells, taking the fungus *C. albicans* as a model system. Initially, as a means to determine that the restriction effect of *C. albicans* to switch its phenotype from yeast to hypha form was due to the presence of the precoating aptamer on yeast, we separated biofilm development into adhesion and growth phases. The result of the transcription assay during the initial stage of biofilm formation indicated that the aptamer involvement in reducing the expression of adhesion and hypha-associated gene ( *ALS3* and *HWP3*) ^20^, and the transcription profile was in line with the increased concentration of the aptamer, but not on *C. albicans* strains. This result suggests that the aptamer (Ca-apt1) might bind to a common chemical structure on the fungal yeast cell, probably glucans and chitins ^21^ that form the basic cell wall scaffold ^22^. We presume that this aptamer may interfere with the adherence mechanism where these molecules are involved ^22^.

Our study is different from most *in vitro* studies, which use an antibody that recognizes cell-wall proteins, that are commonly related to the host immunological status ^23, 24^. In this study, the precoating aptamer (Ca-apt1) does not directly recognize the cell wall proteins as an antibody would. In this way, we assumed that based on the *ALS3*/ *HWP1* profiles explained above, the obvious virulence factors (morphology transformation from yeast to hypha) were more likely to be the main effect of precoating Ca-apt1. This assumption was supported by microscopic observation that at the attachment phase (90 min time point) the morphology of *C. albicans* consisted of both yeast cells and blastospore only. This result confirmed the transcription assay that the aptamer involvement in reducing adhesion and hypha-associated gene ( *ALS3* and *HWP3*) ^20^ was in line with the increased concentration, but not on *C. albicans* strains. Likewise, this qualitative data exhibited a significant potentiate of Ca-apt1 in modulating biofilm-associated genes, as well as *C. albicans* morphogenesis, at the early step of biofilm formation.

Although the pattern was similar, in general, the biofilm formation (90 min time point) treated with CCSC resulted in a higher viable cell than its NCSC counterpart, as we observed that after being treated for 48-h by CCSC or NCSC. Different cellular response of *C. albicans* was noted, both by analysing the gene ( *ALS3* and *HWP1*) profiles and observing microscopically. At this maturation biofilm state, the induction of hypha-associated gene ( *HWP1*) was significantly different, but not for the gene's adhesion ( *ALS3*). We noted that the transcription level of *HWP1* by either *C. albicans* strain was higher than *ALS3* only when the preformed biofilm was treated with CCSC. This result indicates that unlike *ALS3*, the transcription level of *HWP1* was dependent on the source of the condensate smoke used. The effect of condensate smoke on *C. albicans* cells viability in biofilm development does not relate to *C. albicans* strain. We assumed that different tobacco components, specifically the principal component alkaloid of tobacco (nicotine), between kretek and conventional cigarettes ^25, 26^, may have a different effect on *C. albicans* when the fungus was growing in distinct environmental conditions that trigger the hyphal growth. Additionally, the *HWP1* product (Hwp1) is a hypha-association protein commonly expressed on germ tube ^27, 28^. Indeed, this result can be considered after 90 min, when germ tube was induced ^29^. The upregulation of *HWP1* when the biofilm is maturing (48-h) is a survival pathway used by *C. albicans* to resist, or be tolerant against, the effect of chemical toxic-containing CCSC, by modulating the hypha form.

It has been reported that, like NCSC, CCSC comprises a high number of dangerous alkaloid chemical compounds, the most abundant of these being nicotine ^25^. A previous report also showed that the CCSC and NCSC used in this study deliver significant quantities of carbon monoxide (CO), tar and presumably other toxic components of tobacco smoke. Their smoke chemistry were found to be similar ^30^. The other chemical compound in the gas phase of cigarette smoke is nitrogen (N2) ^31^. The nicotine in smoke yields from CCSC is equally delivered as by NCSC ^32^. However, the nitrogen content in CCSC is lower than found in conventional cigarettes/NCSC ^30^. The most distinguished particle-phase, which is only found in CCSC, is eugenol ^15^. Eugenol is a phenylpropanoid compound reported to have antimicrobial activity against planktonic cells of *C. albicans* and sessile cells within *C. albicans* biofilms ^33^.

In contrast to the eugenol, the presence of nicotine in cigarette smoke promotes a high level of adhesins leading to the increased adherence of *C. albicans* ^34^ on a solid surface, as found in this study. Hence, it has the potential to increase biofilm formation by *C. albicans*. Considering that eugenol impairs the growth of *C. albicans*, we assume that under the experimental condition set in this study, *C. albicans* sensed eugenol in CCSC, and the presence of nitrogen helped it to grow. Since we grew the fungus in a hypha conditioning medium, the result obtained in this study indicates that by comparing with NCSC, the lower nitrogen in CCSC is the likely reason for the augmentation of *C. albicans* biofilm formation ^9, 35^ enhanced by the CCSC. As we evaluated microscopically, more hypha in 48-h biofilm was observed in response to the presence of CCSC than NCSC. 

As shown in the literature, cigarette smoke exposure (CSC) has a potential adverse health effect on the oral ecosystem ^36^, and the growth rate of *C. albicans* increases when the fungus is grown in the presence of non-clove cigarette smoke condensate/NCSC ^5^. Indeed, cigarette smoke is an important predisposing factor for oral candidiasis ^35^. Although kretek/clove cigarette smoke condensate (CCSC) and NCSC contains many toxicants, they have some different chemical constituents ^30– 32^, which may lead to the differences in *Candida* cell behaviour that we observed in this study.

We further address whether the precoating aptamer could adversely affect the preformed biofilm formation's viability when treated with CCSC or NCSC. The experiment was done to confirm that, on the one hand, experimental *C. albicans* biofilm formation promoted by cigarette smoke (CCSC and NCSC) could be induced. On the other, that the precoating aptamer is needed for the success of this model. In this way, the discrepancy between CCSC and NCSC on *C. albicans* susceptibility was measured using a MTT reduction assay. This is a reliable test for an indirect method to quantify biofilm cell numbers ^37, 38^. It has been demonstrated that when growing as a biofilm, the metabolic activity of *C. albicans* increases over time ^39^. However, another factor (the aptamer) was added in the current study. Here, we observed that the control group ( *C. albicans* of untreated condensate cigarette smoke/ CSC, without precoating aptamer (Ca-apt1) remained growing as a biofilm throughout the experimental time period set in this study. This result may suggest that *C. albicans* used the tobacco compound as a nutritional source, as aromatic hydrocarbons in cigarette smoke can be converted by the fungus ^35^.

Further, we found that after treating with NCSC for 48-h, the lowest concentration of tested aptamer (1ng/µL) was enough to reduce the cell viability > 50%. This indicates that the effectivity of NCSC as an accelerator of biofilm formation ^5^ was significantly suppressed. Hence, it affirms the anti-adhesion effect of the aptamer on biofilm development accelerated by conventional cigarette smoke ^5, 7^. Surprisingly, aptamer-precoated *C. albicans* behaves differently upon exposure to growth medium-containing CCSC. We noted that at one ng/µL of the aptamer, significant cell growth changes were not observed compared to the control (uncoated aptamer). However, at 10 ng/µL, the aptamer could inhibit the biofilm cell growth until *C. albicans* reached a steady state at a 48-h time period, more and less at a similar rate to those cells exposed by NCSC. The result was supported by the light microscopy data, in which we observed that when the fungus was exposed with the minimal concentration (1 ng/µL) of Ca-apt1, the composition of 48-h-old biofilms consisted of pseudo hypha, almost similar to the control ( *C. albicans* without pre-coated aptamer). We also observed that when the concentration of the tested aptamer was increased by 10-fold, it prevented the cell's viability and successful germination of the adherence cells, resulting in scant biofilms, and more yeast cells were observed microscopically in treated groups (CCSC and NCSC exposure). For NCSC, we observed that regardless of the tested aptamer's concentration in precoating step, the NCSC-treated biofilms were predominantly in the blastospore or yeast form compared to the control. After 48-h exposure, many of these biofilm cells were unbudded and had an unelongated morphology. At the end of the experiment time, both *C. albicans* strains, which belonged to the treated groups ( *C. albicans* with precoating aptamer), showed a similar phenotype response to either CCSC or NCSC exposure. Our result contradicted a previous report that demonstrated that the clinical isolate showed less biofilm growth activity than the laboratory strain ^40^. We reasoned that the similar trend found in either *C. albicans* strain used in this study is probably because the clinical isolate and the ATCC strain used in this study have a similar karyotype as it has been previously reported ^41^.

## Conclusion

Regardless of the mechanism involved, this study demonstrated that both CCSC and NCSC have a potentiate to enhance biofilm formation by *C. albicans*. By exposing either cigarette smoke prepared from clove and non-clove cigarettes, *C*. *albicans*, either clinical isolate or the reference strain, shows a similar trend in its capacity to form biofilm. However, the pattern of biofilm formation was different. Besides, this study clearly indicates that the aptamer (Ca-apt1) had an impact on the degree of cell adhesion and its concentration affected the profile of biofilm formation at the maturity phase. However, the underlying mechanisms remain to be investigated at molecular level. The underlying mechanism is particularly important for pathogenic fungus, such as *C. albicans*, during exposure to tobacco smoke.

## Data availability

### Underlying data

Figshare: Underlying data for ‘The discrepancy between Clove and Non-Clove Cigarette Smoke-Promoted Candida albicans Biofilm Formation with precoating RNA-aptamer’, DOI 10.17605/OSF.IO/WF2KB ^42^

This project contains the following underlying data:

- Data for Figs 2-4 CCSC-2021 F1000 Aptamer Cigarete.xlsx

Data are available under the terms of the Creative Commons Zero "No rights reserved" data waiver (CC0 1.0 Public domain dedication).

License: CC0 1.0 Universal

## References

[1] Sudbery P Gow N Berman J : The distinct morphogenic states of *Candida albicans*. *Trends Microbiol.* 2004;12(7):317–24. 10.1016/j.tim.2004.05.008 15223059

[2] Akpan A Morgan R : Oral candidiasis. *Postgrad Med J.* 2002;78(922):455–9. 10.1136/pmj.78.922.455 12185216 PMC1742467

[3] Soll DR : Candida commensalism and virulence: the evolution of phenotypic plasticity. *Acta Trop.* 2002;81(2):101–10. 10.1016/s0001-706x(01)00200-5 11801217

[4] Whiteway M Bachewich C : Morphogenesis in *Candida albicans*. *Annu Rev Microbiol.* 2007;61:529–53. 10.1146/annurev.micro.61.080706.093341 17506678 PMC4452225

[5] Semlali A Killer K Alanazi H : Cigarette smoke condensate increases *C. albicans* adhesion, growth, biofilm formation, and *EAP1, HWP1* and *SAP2* gene expression. *BMC Microbiol.* 2014;14:61. 10.1186/1471-2180-14-61 24618025 PMC3995653

[6] Alanazi H Semlali A Perraud L : Cigarette smoke-exposed *Candida albicans* increased chitin production and modulated human fibroblast cell responses. *Biomed Res Int.* 2014;2014:963156. 10.1155/2014/963156 25302312 PMC4180399

[7] Gani BA Winiati E Bachtiar BM : The role of cigarettes smoke condensatein enhanced *Candida albicans* virulence of salivary isolates based on time and temperature. *J Int Dent Medical Res.* 2017;10(Specialissue):769–777. Reference Source

[8] Mun M Yap T Alnuaimi AD : Oral candidal carriage in asymptomatic patients. *Aust Dent J.* 2016;61(2):190–5. 10.1111/adj.12335 25912248

[9] Gunasegar S Himratul-Aznita WH : Nicotine enhances the thickness of biofilm and adherence of *Candida albicans* ATCC 14053 and *Candida parapsilosis* ATCC 22019. *FEMS Yeast Res.* 2019;19(2). 10.1093/femsyr/foy123 30476044

[10] Thakur JS Garg R Narain JP : Tobacco use: a major risk factor for non communicable diseases in South-East Asia region. *Indian J Public Health.* 2011;55(3):155–60. 10.4103/0019-557X.89943 22089682

[11] Fithria F Adlim M Jannah SR : Indonesian adolescents' perspectives on smoking habits: a qualitative study. *BMC Public Health.* 2021;21(1):82. 10.1186/s12889-020-10090-z 33413232 PMC7791848

[12] Hardesty JJ Kaplan B Martini S : Smoking among female daily smokers in Surabaya, Indonesia. *Public Health.* 2019;172:40–42. 10.1016/j.puhe.2019.03.007 31158567

[13] Roemer E Dempsey R Schorp MK : Toxicological assessment of kretek cigarettes: Part 1: background, assessment approach, and summary of findings. *Regul Toxicol Pharmacol.* 2014;70 Suppl 1:S2–14. 10.1016/j.yrtph.2014.11.015 25498000

[14] Fongsmut T Deerochanawong C Prachyabrued W : Intraoral candida in Thai diabetes patients. *J Med Assoc Thai.* 1998;81(6):449–53. 9676077

[15] Polzin GM Stanfill SB Brown CR : Determination of eugenol, anethole, and coumarin in the mainstream cigarette smoke of Indonesian clove cigarettes. *Food Chem Toxicol.* 2007;45(10):1948–53. 10.1016/j.fct.2007.04.012 17583404

[16] Bachtiar BM Srisawat C Bachtiar EW : RNA aptamers selected against yeast cells inhibit *Candida albicans* biofilm formation in vitro. *Microbiologyopen.* 2019;8(8): e00812. 10.1002/mbo3.812 30779315 PMC6692556

[17] Bachtiar BM Fath T Widowati R : Quantification and Pathogenicity of *Candida albicans* in Denture-Wearing and Nondenture-Wearing Elderly. *Eur J Dent.* 2020;14(3):423–428. 10.1055/s-0040-1712779 32542630 PMC7440952

[18] Pfaller MA Houston A Coffmann S : Application of CHROMagar Candida for rapid screening of clinical specimens for *Candida albicans*, Candida tropicalis, Candida krusei, and Candida (Torulopsis) glabrata. *J Clin Microbiol.* 1996;34(1):58–61. 10.1128/JCM.34.1.58-61.1996 8748273 PMC228730

[19] Bachtiar EW Bachtiar BM : Effect of cell-free spent media prepared from *Aggregatibacter actinomycetemcomitans* on the growth of *Candida albicans* and *Streptococcus mutans* in co-species biofilms. *Eur J Oral Sci.* 2020;128(5):395–404. 10.1111/eos.12725 32808302

[20] Fan Y He H Dong Y : Hyphae-specific genes *HGC1, ALS3, HWP1,* and *ECE1* and relevant signaling pathways in *Candida albicans*. *Mycopathologia.* 2013;176(5–6):329–35. 10.1007/s11046-013-9684-6 24002103

[21] Garcia-Rubio R de Oliveira HC Rivera J : The Fungal Cell Wall: *Candida, Cryptococcus,* and *Aspergillus* Species. *Front Microbiol.* 2020;10:2993. 10.3389/fmicb.2019.02993 31993032 PMC6962315

[22] Ruiz-Herrera J Elorza MV Valentín E : Molecular organization of the cell wall of *Candida albicans* and its relation to pathogenicity. *FEMS Yeast Res.* 2006;6(1):14–29. 10.1111/j.1567-1364.2005.00017.x 16423067

[23] Masuoka J Wu G Glee PM : Inhibition of *Candida albicans* attachment to extracellular matrix by antibodies which recognize hydrophobic cell wall proteins. *FEMS Immunol Med Microbiol.* 1999;24(4):421–9. 10.1111/j.1574-695X.1999.tb01314.x 10435761

[24] Antoran A Aparicio-Fernandez L Pellon A : The monoclonal antibody Ca37, developed against *Candida albicans* alcohol dehydrogenase, inhibits the yeast *in vitro* and *in vivo*. *Sci Rep.* 2020;10(1):9206. 10.1038/s41598-020-65859-4 32514067 PMC7280234

[25] Gellner CA Reynaga DD Leslie FM : Cigarette Smoke Extract: A Preclinical Model of Tobacco Dependence. *Curr Protoc Neurosci.* 2016;77:9.54.1–9.54.10. 10.1002/cpns.14 27696362 PMC5113292

[26] Malson JL Sims K Murty R : Comparison of the nicotine content of tobacco used in bidis and conventional cigarettes. *Tob Control.* 2001;10(2):181–3. 10.1136/tc.10.2.181 11387541 PMC1747555

[27] Chaffin WL : *Candida albicans* cell wall proteins. *Microbiol Mol Biol Rev.* 2008;72(3):495–544. 10.1128/MMBR.00032-07 18772287 PMC2546859

[28] Nobile CJ Nett JE Andes DR : Function of *Candida albicans* adhesin Hwp1 in biofilm formation. *Eukaryot Cell.* 2006;5(10):1604–10. 10.1128/EC.00194-06 17030992 PMC1595337

[29] Bachtiar EW Bachtiar BM Jarosz LM : AI-2 of *Aggregatibacter actinomycetemcomitans* inhibits *Candida albicans* biofilm formation. *Front Cell Infect Microbiol.* 2014;4:94. 10.3389/fcimb.2014.00094 25101248 PMC4104835

[30] Piadé JJ Roemer E Dempsey R : Toxicological assessment of kretek cigarettes: Part 2: kretek and American-blended cigarettes, smoke chemistry and *in vitro* toxicity. *Regul Toxicol Pharmacol.* 2014;70 Suppl 1:S15–25. 10.1016/j.yrtph.2014.12.001 25497993

[31] Borgerding M Klus H : Analysis of complex mixtures--cigarette smoke. *Exp Toxicol Pathol.* 2005;57 Suppl 1:43–73. 10.1016/j.etp.2005.05.010 16092717

[32] Malson JL Lee EM Murty R : Clove cigarette smoking: biochemical, physiological, and subjective effects. *Pharmacol Biochem Behav.* 2003;74(3):739–45. 10.1016/s0091-3057(02)01076-6 12543240

[33] He M Du M Fan M : In vitro activity of eugenol against *Candida albicans* biofilms. *Mycopathologia.* 2007;163(3):137–43. 10.1007/s11046-007-0097-2 17356790

[34] Baboni FB Barp D de Azevedo Izidoro ACS : Enhancement of *Candida albicans* virulence after exposition to cigarette mainstream smoke. *Mycopathologia.* 2009;168(5):227–35. 10.1007/s11046-009-9217-5 19544010

[35] Soysa NS Ellepola ANB : The impact of cigarette/tobacco smoking on oral candidosis: an overview. *Oral Dis.* 2005;11(5):268–73. 10.1111/j.1601-0825.2005.01115.x 16120112

[36] Arcavi L Benowitz NL : Cigarette smoking and infection. *Arch Intern Med.* 2004;164(20):2206–16. 10.1001/archinte.164.20.2206 15534156

[37] Hawser S : Adhesion of different *Candida* spp. to plastic: XTT formazan determinations. *J Med Vet Mycol.* 1996;34(6):407–10. 10.1080/02681219680000721 8971630

[38] Kuhn DM Balkis M Chandra J : Uses and limitations of the XTT assay in studies of *Candida* growth and metabolism. *J Clin Microbiol.* 2003;41(1):506–8. 10.1128/jcm.41.1.506-508.2003 12517908 PMC149594

[39] Chandra J Mukherjee PK Leidich SD : Antifungal resistance of candidal biofilms formed on denture acrylic in vitro. *J Dent Res.* 2001;80(3):903–8. 10.1177/00220345010800031101 11379893

[40] Alnuaimi AD O'Brien-Simpson NM Reynolds EC : Clinical isolates and laboratory reference *Candida* species and strains have varying abilities to form biofilms. *FEMS Yeast Res.* 2013;13(7):689–99. 10.1111/1567-1364.12068 23927631

[41] Klempp-Selb B Rimek D Kappe R : Karyotyping of *Candida albicans* and *Candida glabrata* from patients with *Candida* sepsis. *Mycoses.* 2000;43(5):159–63. 10.1046/j.1439-0507.2000.00555.x 10948811

[42] Bachtiar EW Gani BA Deviana A : The discrepancy between clove and non-clove cigarette smoke-promoted *Candida albicans* biofilm formation with precoating RNA-aptamer.2021. 10.17605/OSF.IO/WF2KB PMC8311812 34367616

