# Supplementary material for: The Discrepancy between Clove and Non-Clove Cigarette Smoke-Promoted Candida albicans Biofilm Formation with Precoating RNA-aptamer
Source: F1000Res. 2021 Jul 29;10:372. Originally published 2021 May 11. [Version 3] doi: 10.12688/f1000research.52266.3 (PMC8311812; doi:10.12688/f1000research.52266.3)
Supplement: Peer review history

# Peer review: Reviewer response for version 3

**DOI:** 10.5256/f1000research.58969.r90712

**Published:** 2021-08-23

**Authors:** Zamirah Zainal-Abidin

**Affiliations:** 1 Department of Craniofacial Diagnostics and Biosciences, Faculty of Dentistry, Universiti Kebangsaan Malaysia, Kuala Lumpur, Malaysia

There are still some comments from the second review yet to be addressed by the authors: 

1. Abstract: In the sentence "Interestingly, we noted that the enhancer effect of treated CCSC..." the word 'treated' is still not replaced with 'added' since the CCSC was not treated in this study.
2. Introduction: In paragraph 3, the word 'strain' should be replaced with 'strand'. The term 'strain' is not used to describe DNA and RNA.
3. Methods: In the final sentence of the subheading ' Preparation of cigarette smoke condensates', please specify what is being 'kept at 4°C until use.'

I hope the authors will address the above comments. Thank you.

Is the work clearly and accurately presented and does it cite the current literature?

Yes

If applicable, is the statistical analysis and its interpretation appropriate?

Yes

Are all the source data underlying the results available to ensure full reproducibility?

Yes

Is the study design appropriate and is the work technically sound?

Yes

Are the conclusions drawn adequately supported by the results?

Yes

Are sufficient details of methods and analysis provided to allow replication by others?

Yes

Reviewer Expertise:

Pharmacology, Oral Microbiology

I confirm that I have read this submission and believe that I have an appropriate level of expertise to confirm that it is of an acceptable scientific standard.

# Peer review: Reviewer response for version 2

**DOI:** 10.5256/f1000research.58397.r89419

**Published:** 2021-07-22

**Authors:** Samira Shirooie

**Affiliations:** 1 Pharmaceutical Sciences Research Center, Health Institute, Kermanshah University of Medical Sciences, Kermanshah, Iran

I have checked the revised article and the author's responses. In my opinion, the revisions are appropriate.

Is the work clearly and accurately presented and does it cite the current literature?

Yes

If applicable, is the statistical analysis and its interpretation appropriate?

Partly

Are all the source data underlying the results available to ensure full reproducibility?

Yes

Is the study design appropriate and is the work technically sound?

Yes

Are the conclusions drawn adequately supported by the results?

Yes

Are sufficient details of methods and analysis provided to allow replication by others?

Partly

Reviewer Expertise:

Pharmacology

I confirm that I have read this submission and believe that I have an appropriate level of expertise to confirm that it is of an acceptable scientific standard.

# Peer review: Reviewer response for version 2

**DOI:** 10.5256/f1000research.58397.r89420

**Published:** 2021-07-13

**Authors:** Zamirah Zainal-Abidin

**Affiliations:** 1 Department of Craniofacial Diagnostics and Biosciences, Faculty of Dentistry, Universiti Kebangsaan Malaysia, Kuala Lumpur, Malaysia

Almost all comments and suggestions submitted during the initial review were addressed. 

 There are a few minor corrections as follows:

1. Abstract: the word 'treated' is still not replaced with 'added'.
2. Introduction: In paragraph 3, the word 'strain' should be replaced with 'strand'.
3. Methods: In the final sentence, please specify what is being 'kept at 4°C until use.'

That is all, thank you.

Is the work clearly and accurately presented and does it cite the current literature?

Yes

If applicable, is the statistical analysis and its interpretation appropriate?

Yes

Are all the source data underlying the results available to ensure full reproducibility?

Yes

Is the study design appropriate and is the work technically sound?

Yes

Are the conclusions drawn adequately supported by the results?

Yes

Are sufficient details of methods and analysis provided to allow replication by others?

Yes

Reviewer Expertise:

Pharmacology, Oral Microbiology

I confirm that I have read this submission and believe that I have an appropriate level of expertise to confirm that it is of an acceptable scientific standard.

# Peer review: Reviewer response for version 1

**DOI:** 10.5256/f1000research.55516.r87553

**Published:** 2021-06-30

**Authors:** Zamirah Zainal-Abidin

**Affiliations:** 1 Department of Craniofacial Diagnostics and Biosciences, Faculty of Dentistry, Universiti Kebangsaan Malaysia, Kuala Lumpur, Malaysia

This article reported a study on the effects of two types of cigarette smoke condensates (CSC), namely the clove CSC (CCSC) and non-clove CSC (NCSC) on the formation of biofilm **by *C.* *albicans* from clinical isolate and ATCC 10231. This study also investigated the effects of pre-coating RNA aptamer, Ca-apt1 in modulating the gene expression and phenotypic changes of *C.* *albicans* cells that have been exposed to the abovementioned types of SCC, with the result of modulation is concentration-dependent.

 The authors found that *C. albicans *cells of both clinical isolates and ATCC strains, precoated with different concentrations (1 ng/µL and 10 ng/µL) of aptamer (Ca-apt1) showed downregulation of adhesion-associated genes, i.e., *ALS3 *and *HWP1*, where the relative fold changes of the expression of the studied genes were Ca-apt1 concentration-dependent *.* This finding was substantiated by a microscopic study of the formation of the biofilm by C.** *albicans*, compared with the ones without Ca-apt1. 

 The same approach of the investigation was adopted to investigate whether Ca-apt1 confers a protective effect on the CCSC and NCSC induced biofilm formation by *C. albicans*. The results demonstrated that CCS-induced biofilm formation was mitigated in the presence of Ca-apt1. The authors proposed that Ca-apt1 modulates the morphogenesis of *C.* *albicans* growing as a biofilm, as indicated by the predominance of blastospores in the treated Ca-apt1 biofilm, relative to the untreated control. 

 Since adhesion is the crucial step in biofilm formation, the authors suggested that the C-apt1 may bind to a common chemical structure of the *C. albicans* cells that may play a vital role in the adherence of the fungus to a substrate. This suggestion is indeed intriguing, which warrants further studies on the anti-adhesion mechanism of Ca-apt1.

 The most specific comments based on the sections in the manuscript are as follows:

**Title:**

 Capitalization of the title needs to be consistent.

**Abstract:**

 In the sentence “Interestingly, we noted that the enhancer effect of treated CCSC was no longer effective…”. As described in the Methods section, the CCSC did not undergo any form of treatment in this study. It is recommended to rephrase this sentence to present the actual form of the CCSC used in this study.

**Results:**

 1. The first subheading should be reporting on *ALS3* and *HWP1* instead of *HWP1* and *YWP1*.

 2. For Figures 2A and B, it is recommended that the Y-axes be standardized in both graphs, with a maximum relative fold change = 1.0. The comparison between the two strains in terms of inhibition of biofilm formation may then be emphasized. The indication of the dual asterisk (**) in the graphs should also be described. In the subtitle, use 1 ng/µL and 10 ng/µL rather than 1% and 10%. The authors may also describe Figure 2C-E in terms of comparison to the abundance of adherent C. *albicans* cells attached to the surface between the 3 treatments.

 3. In Figure 4, it is recommended that the Y-axes are standardized in both graphs, with a maximum percentage of control = 100. The comparison between the two strains in terms of reduction of the pre-formed biofilm can then be highlighted.

**Discussion:**

 1. In paragraph 4, in this study, the comparison in terms of chemical contents between NCSC and CCSC is not reported. The concentrations of the common chemical compounds may be different among cigarette manufacturers. Therefore, it is recommended to provide relevant information on the concentrations of CSC chemicals in cigarettes used specifically for this study.

 2. In the final paragraph, the statement “Hence, it affirms that the anti-adhesion effect of the aptamer on biofilm development is enhanced by conventional cigarette smoke”. The authors should point out the results in this study or cite references that compared the formation of *C.* *albicans* biofilms without CSC to support that statement.

 3. In the statement “...with the minimum concentration (1 ng/μL) of CCSC...”, it is supposed to be aptamer.

**Conclusion:**

 In the statement “… *C. albicans*, either clinical isolate or the reference strain, shows a similar trend.” The authors will need to elaborate a little more on what trend they are referring to e.g., gene expression?

**Other comments:**

 The manuscript is well written, with minor grammatical errors and some typos, which can be easily corrected by giving it a manuscript editing round-up.

 In general, the authors have shown in this study the potential of Ca-apt1, an RNA aptamer in modulating virulence factors of *C. albicans*, one of the opportunist microorganisms within the oral cavity. In addition, the authors also have shown that cigarette smoke condensates can exacerbate the growth of *C.* *albicans* biofilms and how adding Ca-apt1 has mitigated this effect. I hope the authors will further investigate the underlying protective mechanism of aptamer at both genotypic and phenotypic levels, against the exacerbation of biofilm formation and pathogenicity of *C.* *albicans* when they are exposed to cigarette smoke, as well as adopt a tissue culture or animal model appropriate for investigation.

Is the work clearly and accurately presented and does it cite the current literature?

Yes

If applicable, is the statistical analysis and its interpretation appropriate?

Yes

Are all the source data underlying the results available to ensure full reproducibility?

Yes

Is the study design appropriate and is the work technically sound?

Yes

Are the conclusions drawn adequately supported by the results?

Yes

Are sufficient details of methods and analysis provided to allow replication by others?

Yes

Reviewer Expertise:

Pharmacology, Oral Microbiology

I confirm that I have read this submission and believe that I have an appropriate level of expertise to confirm that it is of an acceptable scientific standard.

# Response

**Published:** 2021-07-03

**Authors:** Boy Muchlis Bachtiar

**Affiliations:** Universitas Indonesia, Indonesia

**Reviewer # 2**

 The most specific comments based on the sections in the manuscript are as follows:

 Title:

 Capitalization of the title needs to be consistent.

 Author’s response ( **AR**):We agree. Thank you. As suggested, we have corrected the capitalization of the title.

 Abstract:

 In the sentence “Interestingly, we noted that the enhancer effect of treated CCSC was no longer effective…”. As described in the Methods section, the CCSC did not undergo any form of treatment in this study. It is recommended to rephrase this sentence to present the actual form of the CCSC used in this study.

**AR**: Thank you for this important correction. Hence, we changed the word to make clear the meaning, as follows;

 “…we noted that the enhancer effect of added CCSC was no longer effective when the yeast had been precoated with 10 ng/μL aptamer tested”.

 3. Results:

 3.1. The first subheading should be reporting on *ALS3* and *HWP1* instead of *HWP1* and *YWP1*.

**AR**: Yes, you right. Thank you. We have changed the name of the gene, as suggested.

 3.2. For Figures 2A and B, it is recommended that the Y-axes be standardized in both graphs, with a maximum relative fold change = 1.0. The comparison between the two strains in terms of inhibition of biofilm formation may then be emphasized. The indication of the dual asterisk (**) in the graphs should also be described. In the subtitle, use 1 ng/µL and 10 ng/µL rather than 1% and 10%. The authors may also describe Figure 2C-E in terms of comparison to the abundance of adherent *C. albicans* cells attached to the surface between the 3 treatments.

**AR**: As suggested by the reviewer, we have standardized the Y-axes in both graphs, where the maximum relative fold change has been set to 1. Moreover, as corrected by the reviewer#1, the dual asterisks were removed. The typos in the subtitle, 1 and 10%, has been changed to 1 and 10 ng/ µL. Lastly, we described the figure 2C-E, where the abundance of adherent *C. albicans* between the 3 treatments was compared. Thank you.

 3.3. In Figure 4, it is recommended that the Y-axes are standardized in both graphs, with a maximum percentage of control = 100. The comparison between the two strains in terms of reduction of the pre-formed biofilm can then be highlighted.

**AR:** yes. We agree. Accordingly, we changed the T-axes in both graphs with a maximum percentage of the control set at 100. Thank you.

 4. Discussion:

 4.1. In paragraph 4, in this study, the comparison in terms of chemical contents between NCSC and CCSC is not reported. The concentrations of the common chemical compounds may be different among cigarette manufacturers. Therefore, it is recommended to provide relevant information on the concentrations of CSC chemicals in cigarettes used specifically for this study.

**AR**: Thank you for the recommendation, and we agree. Thus, as suggested, in paragraph 4, we added a relevant information regarding the smoke chemical in CCSC and NCSC used in this study.

 4.2. In the final paragraph, the statement “Hence, it affirms that the anti-adhesion effect of the aptamer on biofilm development is enhanced by conventional cigarette smoke”. The authors should point out the results in this study or cite references that compared the formation of *C. albicans* biofilms without CSC to support that statement.

**AR**: We agree, and we have revised the sentence, and added references needed. It can be seen in the revised version of this manuscript; the revised sentence as follows:

 “Hence, it affirms that the anti-adhesion effect of the aptamer on biofilm development is enhanced accelerated by conventional cigarette smoke ^5, 7^.” Thank you.

 4.3. In the statement “...with the minimum concentration (1 ng/μL) of CCSC...”, it is supposed to be aptamer.

**AR**: We agree, and we have corrected the typo. Thank you.

 5. Conclusion:

 In the statement “… *C. albicans*, either clinical isolate or the reference strain, shows a similar trend.” The authors will need to elaborate a little more on what trend they are referring to e.g., gene expression?

**AR:** We agree. Thus, we revised the sentence to elaborate the meaning of “trend” specifically. Hence, the new sentence is now becoming : “...shows a similar trend in its capacity to form biofilm.”

 6. Other comments:

 The manuscript is well written, with minor grammatical errors and some typos, which can be easily corrected by giving it a manuscript editing round-up. In general, the authors have shown in this study the potential of Ca-apt1, an RNA aptamer in modulating virulence factors of *C. albicans*, one of the opportunist microorganisms within the oral cavity. In addition, the authors also have shown that cigarette smoke condensates can exacerbate the growth of *C. albicans* biofilms and how adding Ca-apt1 has mitigated this effect. I hope the authors will further investigate the underlying protective mechanism of aptamer at both genotypic and phenotypic levels, against the exacerbation of biofilm formation and pathogenicity of *C. albicans* when they are exposed to cigarette smoke, as well as adopt a tissue culture or animal model appropriate for investigation.

**AR**: Thank you. Hence, we added the reviewer’s suggestion at the end of the conclusion.

# Peer review: Reviewer response for version 1

**DOI:** 10.5256/f1000research.55516.r86552

**Published:** 2021-06-29

**Authors:** Samira Shirooie

**Affiliations:** 1 Pharmaceutical Sciences Research Center, Health Institute, Kermanshah University of Medical Sciences, Kermanshah, Iran

In the introduction, mention the genes ALS3 & HWP1 and their roles.

 In results, in the first headline was written "Evaluation of the mRNA levels of *HWP1* and ***YWP1* **in pre-coating aptamer *C. albicans" *that must be corrected to "Evaluation of the mRNA levels of *HWP1* and ***ALS3 ***in pre-coating aptamer *C. albicans" .*

 The data of the control group should be shown in figures 2A and 2B.

 Write abbreviations in separate part before introduction.

 In figures 3 A & B, compare the CCSC and NCSC groups with their control group not with the another control group, for example compare CCSC for HWP1 expression level with the control group of HWP1 not with ALS3.

Is the work clearly and accurately presented and does it cite the current literature?

Yes

If applicable, is the statistical analysis and its interpretation appropriate?

Partly

Are all the source data underlying the results available to ensure full reproducibility?

Yes

Is the study design appropriate and is the work technically sound?

Yes

Are the conclusions drawn adequately supported by the results?

Yes

Are sufficient details of methods and analysis provided to allow replication by others?

Partly

Reviewer Expertise:

Pharmacology

I confirm that I have read this submission and believe that I have an appropriate level of expertise to confirm that it is of an acceptable scientific standard, however I have significant reservations, as outlined above.

# Response

**Published:** 2021-07-03

**Authors:** Boy Muchlis Bachtiar

**Affiliations:** Universitas Indonesia, Indonesia

**Reviewer #1**

 1. In the introduction, mention the genes ALS3 & HWP1 and their roles.

 Author’s response ( **AR**): We have mentioned the suggestion, and it can be seen at the end of paragraph one of the introduction section, as follows;

... *In this regard, the presence of nicotine in cigarette smoke* *promotes C. albicans growth rate and adhesion, in which, a higher level of expression of genes related to adherence, such as ALS3 and HWP1 have been reported^9^*.

 2. In results, in the first headline was written "Evaluation of the mRNA levels of *HWP1* and ***YWP1* **in pre-coating aptamer *C. albicans" *that must be corrected to "Evaluation of the mRNA levels of *HWP1* and ***ALS3 ***in pre-coating aptamer *C. albicans.*

**AR**: Yes, you right. Thank you. Accordingly, we have corrected the name of the gene.

 3. The data of the control group should be shown in figures 2A and 2B.

**AR**: As suggested, the control groups have been added in figures 2A and 2B. Thank you.

 4. Write abbreviations in separate part before introduction.

**AR**: We only use abbreviations or acronym for they have been widely known in scientific or academic communication.

 5. In figures 3 A & B, compare the CCSC and NCSC groups with their control group not with another control group, for example compare CCSC for HWP1 expression level with the control group of HWP1 not with ALS3.

**AR**: Thank you for this important suggestion. As suggested, we have deleted the double asterisks, as it can be confusing. Thank you.
