# Supplementary material for: The Discrepancy between Clove and Non-Clove Cigarette Smoke-Promoted Candida albicans Biofilm Formation with Precoating RNA-aptamer
Source: F1000Res. 2021 Jul 12;10:372. Originally published 2021 May 11. [Version 2] doi: 10.12688/f1000research.52266.2 (PMC8311812; doi:10.12688/f1000research.52266.2)
Supplement: Peer review history

# Response

**Published:** 2021-07-03

**Authors:** Boy Muchlis Bachtiar

**Affiliations:** Universitas Indonesia, Indonesia

**Reviewer #1**

 1. In the introduction, mention the genes ALS3 & HWP1 and their roles.

 Author’s response ( **AR**): We have mentioned the suggestion, and it can be seen at the end of paragraph one of the introduction section, as follows;

... *In this regard, the presence of nicotine in cigarette smoke* *promotes C. albicans growth rate and adhesion, in which, a higher level of expression of genes related to adherence, such as ALS3 and HWP1 have been reported* *^9^*.

 2. In results, in the first headline was written "Evaluation of the mRNA levels of *HWP1* and ***YWP1* **in pre-coating aptamer *C. albicans" *that must be corrected to "Evaluation of the mRNA levels of *HWP1* and ***ALS3 *** in pre-coating aptamer *C. albicans.*

**AR**: Yes, you right. Thank you. Accordingly, we have corrected the name of the gene.

 3. The data of the control group should be shown in figures 2A and 2B.

**AR**: As suggested, the control groups have been added in figures 2A and 2B. Thank you.

 4. Write abbreviations in separate part before introduction.

**AR**: We only use abbreviations or acronym for they have been widely known in scientific or academic communication.

 5. In figures 3 A & B, compare the CCSC and NCSC groups with their control group not with another control group, for example compare CCSC for HWP1 expression level with the control group of HWP1 not with ALS3.

**AR**: Thank you for this important suggestion. As suggested, we have deleted the double asterisks, as it can be confusing. Thank you.
